# Change in BMI and Fitness among Primary School Children in Austria: A 24-Month Follow-Up Study of 303 Children Measured before and during the Ongoing COVID-19 Pandemic

**DOI:** 10.3390/sports10050078

**Published:** 2022-05-19

**Authors:** Gerald Jarnig, Reinhold Kerbl, Mireille N. M. van Poppel

**Affiliations:** 1 Institute of Human Movement Science, Sport and Health, University of Graz, 8010 Graz, Austria; mireille.van-poppel@uni-graz.at; 2 Department of Pediatrics and Adolescent Medicine, LKH Hochsteiermark, 8700 Leoben, Austria; reinhold.kerbl@kages.at

**Keywords:** COVID-19, children, school, body mass index, cardiorespiratory endurance, standing long jump, shuttle run, flexibility, reaction time, full body coordination, fitness, health-related fitness, primary school

## Abstract

The ongoing COVID-19 pandemic not only impacted the health of school children directly through SARS-CoV-2 infections, but the associated closures of schools and sports facilities also resulted in long-term negative side effects. The aim of this study was to investigate the effects of COVID-19-related mitigation measures on the health and fitness status of primary school children in Austria. A total of 303 primary school children participated in the longitudinal study. Data on height, weight, and fitness were collected before the COVID-19 pandemic (September 2019) and at one-year intervals (September 2020 and September 2021) during the course of the pandemic. In the first year, from September 2019 to September 2020, there were alarming increases in body mass index (BMI) standard deviation scores (SDSs) (from 0.32 to 0.49) and dramatic decreases in both cardiorespiratory endurance (CRE) (from 0.49 to −0.43) and action speed (from −0.31 to −0.64). In the second year (September 2020 to September 2021), the BMI scores stabilized, and improvements in CRE were observed, especially in the subgroup of children who were members of sports clubs. In the future, more initiatives and projects, in addition to sports club activities, should be started and expanded, particularly in schools, to specifically counteract the observed health damage and, thus, have a positive effect on the development of all children, especially those without sports club membership.

## 1. Introduction

The COVID-19 pandemic changed the living situation for children and adolescents worldwide [1,2]. Schools, sports clubs, and leisure activities were closed or prohibited, and children remained isolated with their families at home for long periods of time. Many studies report dramatic changes in children’s eating habits, screen time, sleep time, and physical activity behaviors [3,4,5,6,7,8,9,10,11]. Studies also report increased mental health problems in children and adolescents [12,13,14].

The restrictions caused by the COVID-19 pandemic had an equally significant impact on the health and fitness of children and adolescents [15,16,17]. In particular, cardiorespiratory fitness (CRF), which is a strong health maker [18], and changes in body mass index (BMI) showed dramatic negative trends due to the COVID-19 restrictions [16,19].

There are already a large number of studies reporting body mass index changes in children and adolescents [16,19,20,21] but only a few reports on the effects of COVID-19 restrictions on endurance, strength, and speed [15,17,22,23,24,25]. Furthermore, to date, no study has reported the effects of the COVID-19 pandemic on BMI or various fitness parameters over a 2-year observation period.

The aim of this study was to report on changes in the BMI and various fitness parameters of primary school children during the ongoing COVID-19 pandemic over a 24-month period and to investigate whether there were different trends in subgroups according to sex (girls/boys) or sports club membership (yes/no). 

## 2. Materials and Methods

### 2.1. Design

This study was originally designed as a randomized controlled trial to investigate the effects of a physical education intervention, using a newly created Austrian Fitness Monitoring System for School Children (AUT FIT) [26]. Baseline measurements were carried out in September 2019. However, due to COVID-19 restrictions, the intervention had to be halted. Follow-up fitness measurements, which included all baseline tests and had comparable testing conditions, were able to be carried out under strict hygiene regulations, at one-year time intervals, in September 2020 and September 2021. The study was registered in the German Clinical Trials Registry (ID DRKS00023824) and approved by the Research Ethics Committee of the University of Graz, Styria, Austria (GZ. 39/23/63 ex 2018/19).

### 2.2. Selection of Schools and Participations

**Figure 1:**
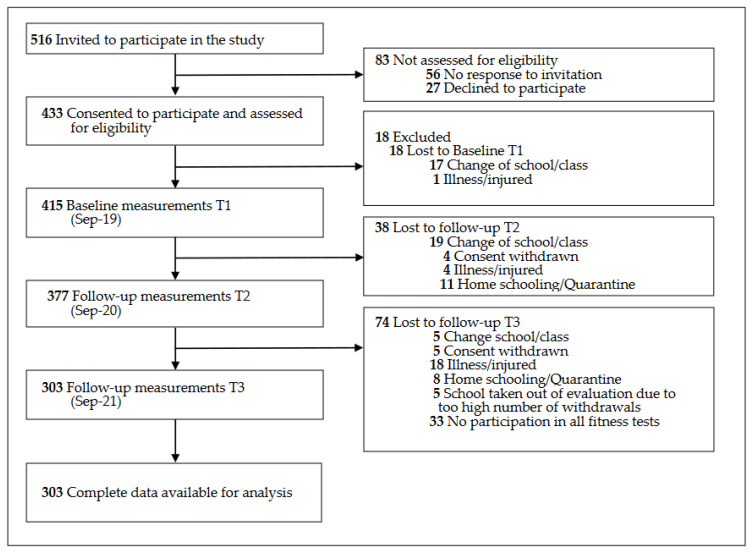
Flow diagram.

A random-number generator was used to select 12 out of 39 primary schools in the district of Klagenfurt (Austria). Inclusion criteria were that children participating in the study had no physical limitations and were attending the second or third grade of primary school at the time of the baseline measurements. In the spring of 2019, all children who were in first or second grade in one of the 12 schools at that time were invited to participate in the study. As the children in the second grade had left school in 2021 and were, thus, not available for the 2-year follow-up measurements, we restricted the analysis in this paper to the 516 children who were in first grade at baseline. A total of 433 (83.9%) legal guardians of those children gave written consent for their children to participate (Figure 1) and indicated, in writing, whether or not their child had a sports club membership.

### 2.3. Procedures

Data collection was conducted by a team of sports scientists and physical education teachers who were specially trained to conduct the test. Baseline measurements (T1) were carried out in September 2019, followed by the start of a sports intervention. The curriculum’s focus and number of physical education lessons in both intervention and control groups were the same; the only difference between them was that, in the intervention group, the physical education lessons were led by a teacher specially trained in physical education and sport. In March 2020, the sport intervention had to be interrupted due to the worldwide spread of the COVID-19 pandemic. After the outbreak of the COVID-19 pandemic and associated school closures, the intervention was discontinued. Follow-up measurements were performed in June 2020, September 2020, March 2021, June 2021, and September 2021. Due to COVID-19, only the fitness tests performed in September 2020 (T2) and September 2021 (T3) were carried out under conditions comparable to the baseline measurements in September 2019 (T1), and, in addition, some of the norm values were only available in year steps. Therefore, only data from the September 2019 (T1, before COVID-19), September 2020, and September 2021 (T2 and T3, after COVID-19) test phases were used in this paper.

To illustrate the government-imposed restrictions over the period of interest, the Oxford COVID-19 Government Response Tracker (OxCGRT) provides the internationally comparable stringency level for Austria (Supplementary Table S1 and Figure S1). Based on Austrian legal regulations, which can be viewed in the Federal Law Gazette [27], a more detailed self-developed stringency level for primary school children was developed, showing the effects of pandemic mitigation on children over time (Supplementary Table S1 and Figure S2).

### 2.4. Outcomes

The primary outcome of this study was the impact of COVID-19-related school closures and physical activity restrictions (sports facility and playground closures) on the fitness and health status of primary school children.

Secondary analyses were conducted for subgroups by sex and sports club membership.

#### 2.4.1. Anthropometric Data

Anthropometric data included height (cm) and weight (kg). The height of the children was measured to the nearest 0.1 cm, using a portable stadiometer (SECA 213, Hamburg, Germany). Weight was measured to the nearest 0.1 kg, using an electronic scale (BOSCH PPW4202/01, Nürnberg, Germany). Body mass index (BMI) was calculated as weight in kilograms divided by height in meters, squared.

#### 2.4.2. Physical Fitness

- Cardiorespiratory Endurance

A 6-min run (6MR) was used to measure cardiorespiratory endurance (CRE) [28]. Children were instructed to run as far as possible in 6 min. The test was performed in playgrounds, sports fields, and indoor sports halls of schools. A field (6 × 18 m) was marked out with sports poles, and then the four corner poles were moved 0.5 m inward. The children were required to run around the marked field. A group of 6 or 7 children performed the test simultaneously. The distance run by the children was measured in meters.

- Strength (Legs and Arms)

The standing long jump (SLJ) was performed to assess lower body muscle strength [28]. From a baseline, children were asked to jump as far as possible with both legs, and the shortest distance between the baseline and the place where the child’s body (arms or legs) came into contact with the ground was measured with a tape measure to the nearest centimeter. A total of three scoring attempts were made, and the longest jump was included in the overall assessment.

A 1 kg medicine ball throw (MB1kg) was used to measure arm strength [29]. The children held a 1 kg medicine ball, with both hands in front of their chest, with the ball touching the body at the level of the sternum. From a baseline, the medicine ball was thrown forward as far as possible with both hands. The shortest distance between the baseline and the place where the ball made contact with the ground was measured with a tape measure to the nearest centimeter. A total of two scoring attempts were made, and the farthest throw was included in the overall assessment.

- Action Speed

A shuttle run test (4 × 10 SHR) was used to assess the action speed of the children [30]. Two lines (baseline and turning line) were marked on the floor 10 m apart. One easy-to-grab object (O1) was placed 40 cm in front of the baseline, and two objects (O2 and O3), also easy-to-grab, were placed 40 cm behind the turning line. The children had to run from the baseline across the turning line, pick up O2, run back across the baseline, and place O2 down. Then they had to pick up O1, run again across the turning line, place O1 down, pick up O3, and run with it across the baseline. The children were instructed to finish this challenge as quickly as possible. The time was measured with a stopwatch to the nearest 0.01 s. A total of two scoring attempts were made, and the fastest attempt was included in the overall assessment.

- Whole-Body Coordination

Side jumping (JS) was used to assess the children’s whole-body coordination [31]. An area (100 × 50 cm) was marked on the floor, using marking tape, and divided into two squares (Q1 = 50 × 50 cm, Q2 = 50 × 50 cm). At the start, the children stood with both feet in the middle of Q1 and, after the starting command, jumped for 15 s, using both legs, from side to side, between Q1 and Q2. The task was to make as many jumps as possible without touching the markers or jumping out of the square. If a child touched the markers or jumped out of the marked area, that jump was not counted. The number of successful jumps was documented, and each jump across the midline was counted as one jump attempt. Each child had two scoring attempts, and the average of the number of valid jumps from both scoring attempts was used for the overall assessment.

- Flexibility

Flexibility was measured by using the sit-and-reach test. The V sit-and-reach test (VSR) was chosen. For this test, a tape scale was fixed to the floor, and marker tape was used to mark a heel line normal to the tape scale. Children were instructed to sit on the floor with their legs extended, feet spread 30 cm apart, knee joints maximally extended, and heels touching the heel line. The children placed their hands on top of each other and slowly stretched forward as far as possible. The distance between the heel line and the maximum position reached with the fingertips that could be held for two seconds was noted. A total of two scoring attempts were made, and the maximum attempt was scored. To use the reference values of the classical sit-and-stretch test, 23 cm was added to the scoring attempt [32,33,34].

- Reaction Speed

Reaction speed was measured by using the ruler drop test (RD) [35]. The test instructor held a ruler, and the children had to stretch out their hand and form an angle of 45 degrees between their thumb and fingers. The ruler was held centrally by the test instructor, and the zero point was held at the level of the bottom of the thumb. The test instructor dropped the ruler within three seconds of the command “READY”. The distance in centimeters that the ruler fell was recorded. A total of five scoring trials were performed, with the best and worst trials dropped from the score, and the average score calculated from the remaining three trials and included in the overall assessment.

### 2.5. Standardization

Continuous variables were reported as the mean (M) and standard deviation (SD), and categorical variables were reported as the absolute value (n) and percentage (%) for descriptive statistics. No imputation of the data was performed.

Standard deviation scores (SDSs) were calculated for BMI, as well as for the results of the fitness tests. National reference values could not be expressed for BMI (EQUI BMI_AUT_ [36]) in SDS, or were not available for fitness tests; therefore, age- and sex-specific international reference values were used for the calculation of standard deviation scores (SDS) [28,30,37,38] and traditional z-scores (z-values) [29,31,35].

#### 2.5.1. Anthropometric Data

The International Obesity Taskforce (IOTF) [38] reference values were used, and calculations based on the LMS method [39] were performed to calculate the standard deviation scores (in this paper, they are referred to as BMI SDS_IOTF_).

#### 2.5.2. Physical Fitness

For the 6MR and the SLJ, German norm values from the Düsseldorf model ([28] collected 2011–2018) were used; for the 4 × 10 SHR, Portuguese norm values [30] from the Motor Competence Assessment (MCA) of 2019 were used; and for the VSR, Macedonian norm values [37] from the MAKFIT study of 2018 were used. 

For these international references, calculations were performed by using the LMS method [39] with the German (DüMo) [28], Portuguese (MCA) [30], and Macedonian (MAKFIT) [37] reference tables. 

For other fitness tests, z-scores were calculated by using a traditional z-score normalization of different German reference values. For the MB1kg, norm values of the Karlsruhe Test System for Children (KATS-K) [29] from 2001 were used for the JS; norm values of the Motric Module [40] from 2004 were used; and for the RD tests, norm values of Fetz and Kornexl [35] from 1978 were used.

#### 2.5.3. Changes over Time

The changes in BMI SDS, 6MR SDS, SLJ SDS, MB1kg z-value, 4 × 10 SHR SDS, JS z-value, VSR SDS, and RD z-value were analyzed over the observation period, using mixed design analyses of variance (ANOVAs) for sex; sports club membership; and the time points T1, T2, and T3. Harley’s F-max test was used to test for homogeneity. The Greenhouse–Geisser adjustment was used to correct sphericity violations. For the ANOVAs, partial eta squared (η_p_^2^) was used to determine the size of the effect (≥0.01 = small, ≥0.06 = medium, ≥0.14 = large) [41], and only effects that were small or above were considered relevant. 

All tests were two-tailed, with a *p*-value < 0.05 considered statistically significant. Bonferroni correction was used for post hoc tests.

For better visualization and comparability of the various changes of SDS and z-values of BMI and the different fitness tests over time, all SDS and z-values of T1 were set to 0, and change values (∆ SDS and ∆ z-values) were calculated for the periods T1–T2 and T1–T3 for BMI SDS, 6MR SDS, SLJ SDS, MB1kg z-value, 4 × 10 SHR SDS, JS z-value, VSR SDS, and RD z-value.

All statistical calculations were performed by using SPSS Version 27 (IBM Corp. Released 2020. IBM SPSS Statistics for Windows, Armonk, NY, USA: IBM Corp).

## 3. Results

In September 2019, 415 children participated in the baseline measurements. A total of 112 children did not participate in all three measurement time points and were excluded from the analyses. In total, complete anthropometric and fitness data were available for 303 children (Figure 1). The included study population and the group lost to follow-up were compared on the variables of age, sex, sports club membership, BMI, and fitness. Children who could not be followed up had poorer results in the 6MR, SLJ, and 4 × 10 SHR and better results in the RD test, but no differences were found in the anthropometric data (Supplementary Table S2).

In the sample included in the analyses, the mean age at baseline was 7.7 ± 0.4 years (range: 7.1 to 8.9 years), 147 (48.5%) were girls, and 133 (43.9%) children were members of a sports club (Table 1).

### 3.1. Change in BMI

**Figure 2:**
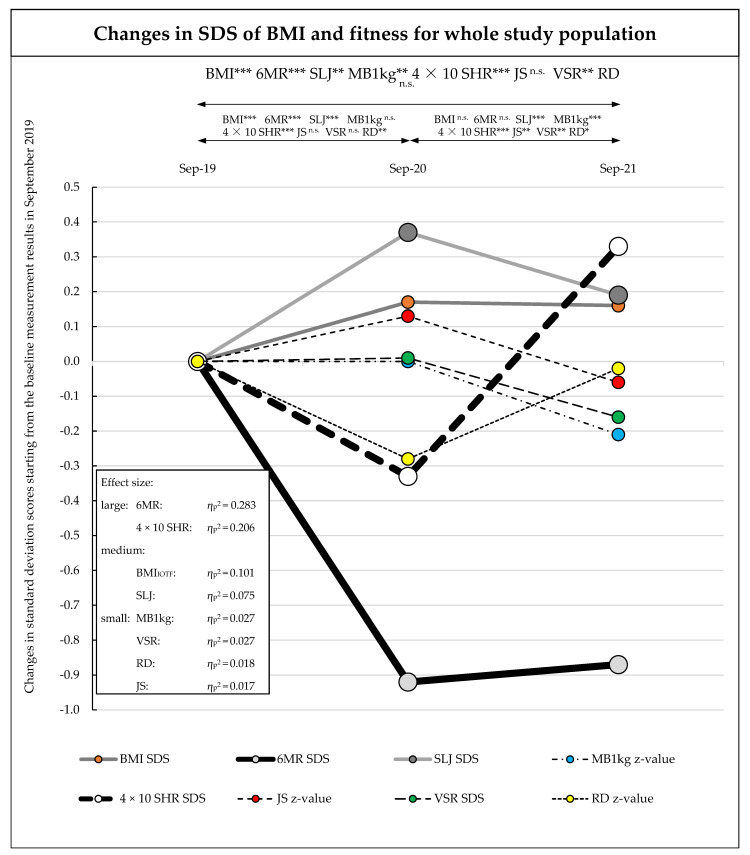
Changes in standard deviation scores of anthropometrics and fitness, starting from the baseline measurement results in September 2019 (T1) via the September 2020 (T2) and September 2021 (T3) test phases. SD = standard deviation; SDS = standard deviation score, z-value = traditional z-score standardization; 6MR = 6-min run, SLJ = standing long jump, MB1kg = medicine ball throw (1 kg), 4 × 10 SHR = 4 m × 10 m shuttle run, JS = jumping sideways, VSR = V sit-and-reach test, RD = ruler drop test; T1 = baseline measurements in September 2019, T2 = follow-up measurements in September 2020, T3 = follow-up measurements in September 2021. Significance level *p*-value: * = *p*-value ≤ 0.05, ** = *p*-value ≤ 0.01, *** = *p*-value ≤ 0.001, n.s. = *p*-value not significant.

**Figure 3:**
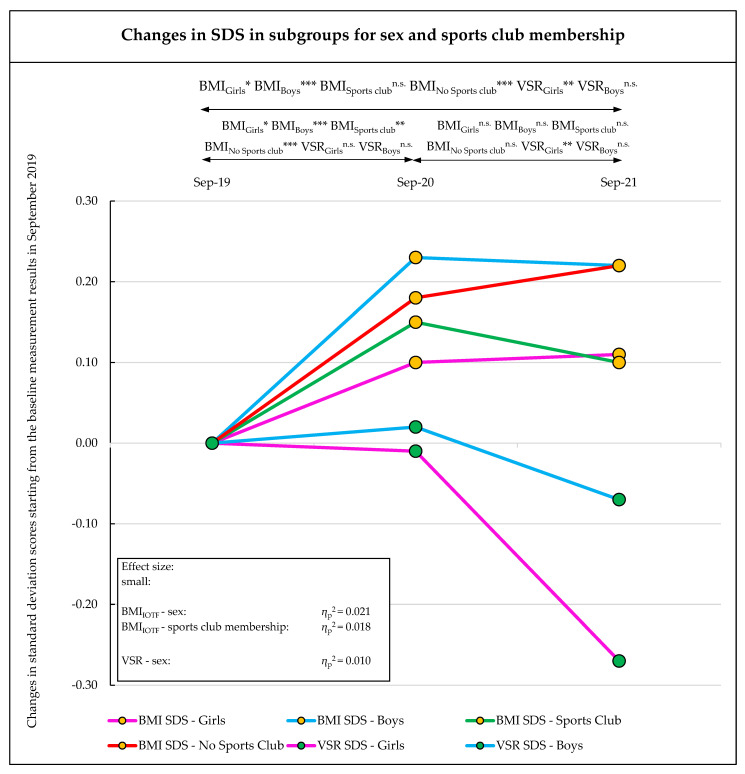
Changes in standard deviation scores of anthropometrics and fitness (only for interaction effects in subgroups), starting from the baseline measurement results in September 2019 (T1) via the September 2020 (T2) and September 2021 (T3) test phases. IOTF = BMI SDS based on International Obesity Taskforce reference centile curves [38], SD = standard deviation; SDS = standard deviation score, VSR = V sit-and-reach test, MAKFIT = Macedonian fitness meter [37], T1 = baseline measurements in September 2019, T2 = follow-up measurements in September 2020, T3 = follow-up measurements in September 2021. Significance level *p*-value: * = *p*-value ≤ 0.05, ** = *p*-value ≤ 0.01, *** = *p*-value ≤ 0.001, n.s. = *p*-value not significant.

Between September 2019 and September 2021, the BMI SDS increased with medium effects over the observation period from 0.32 to 0.48 (main effect time: η_p_^2^ = 0.101; *p* < 0.001), and with small interaction effects over time (time × sex, η_p_^2^ = 0.021; *p* = 0.002; time × sports club, η_p_^2^ = 0.018; *p* = 0.005). The increase mainly took place between September 2019 and September 2020, and no significant changes were detected between September 2020 and September 2021. Between September 2019 and September 2020, boys and children without sports club membership showed a significantly larger increase in BMI SDS (boys, +0.23 (95% CI, 0.17–0.28); no sports club, +0.18 (95% CI, 0.12–0.23)) than girls and children with sports club membership (girls, +0.10 (95% CI, 0.04–0.16); sports club: +0.15 (95% CI, 0.09–0.21)) (Table 1, Table 2 and Table 3, Figure 2 and Figure 3, and Supplementary Tables S3–S5).

### 3.2. Change in Fitness

The cardiorespiratory endurance (6MR) and action speed (4 × 10 SHR) showed large effect changes over the observation period (6MR: T1 to T3: main effect time: η_p_^2^ = 0.283; *p* < 0.001; 4 × 10 SHR: T1 to T3: main effect time: η_p_^2^ = 0.206; *p* < 0.001). No significant differences were found between the sex or sports club membership subgroups (Table 1, Table 2 and Table 3, Figure 2, and Supplementary Tables S3–S5).

Between September 2019 and September 2020, CRE (6MR) showed a significant decrease (T1 = 0.49 to T2 = −0.43 (−0.92 (95% CI, −1.02 to −0.81)); *p* < 0.001) in all subgroups. From September 2020 to September 2021, a small nonsignificant improvement was identified. This improvement was mainly found in boys and children with sports club membership (boys: +0.19 (95% CI, 0.00 to 0.38); *p* = 0.10; sports club: +0.17 (95% CI, −0.06 to 0.39); *p* = 0.46). In contrast, girls and children without sports club membership showed a continued decline in CRE (girls: −0.10 (95% CI, −0.27 to 0.06); *p* > 0.99; no sports club: −0.04 (95% CI, −0.18 to 0.10); *p* > 0.99) (Table 1, Table 2 and Table 3, Figure 2, and Supplementary Tables S3–S5).

Action speed (4 × 10 SHR) showed a dramatic decrease between September 2019 and September 2020 (all: T1 = −0.31 to T2 = −0.64 (−0.33 (95% CI, −0.42 to −0.22)); *p* < 0.001). Between September 2020 and September 2021, a large improvement was evident (all: T2 = −0.64 to T3 = 0.02 (+ 0.66 (95% CI, 0.56 to 0.76)); *p* < 0.001) (Table 1, Table 2 and Table 3, Figure 2, and Supplementary Tables S3–S5).

Leg muscle strength (SLJ) showed a medium effect change over the observation period (T1 to T3: main effect time: η_p_^2^ = 0.075; *p* < 0.001). No significant differences were found between the sex or sports club membership subgroups. There was an improvement in performance of all observed groups between September 2019 and September 2020 (all: T1 = 0.11 to T2 = 0.47 (+ 0.36 (95% CI, +0.26 to +0.46)); *p* < 0.001). In the second year, between September 2020 and September 2021, a decline (all: T2 = 0.47 to T3 = 0.29 (−0.18 (95% CI, −0.26 to −0.10)); *p* < 0.001) in scores was observed in all groups (Table 1, Table 2 and Table 3, Figure 2, and Supplementary Tables S3–S5).

Small effect changes over the observation period were found in arm strength (MB1kg) (T1 to T3: main effect time, η_p_^2^ = 0.027; *p* < 0.001), whole-body coordination (JS) (T1 to T3: main effect time, η_p_^2^ = 0.017; *p* = 0.008), flexibility (VSR) (T1 to T3: main effect time, η_p_^2^ = 0.027; *p* < 0.001), and reaction time (RD) (T1 to T3: main effect time, η_p_^2^ = 0.018; *p* = 0.005). In addition, there were significant differences found in flexibility according to sex subgroup (time * sex: η_p_^2^ = 0.010; *p* = 0.047) (Table 1, Table 2 and Table 3, Figure 2 and Figure 3, and Supplementary Tables S3–S5).

## 4. Discussion

The results of our study showed a major increase in mean BMI-SDS values, mainly during the first year of the observation period (September 2019 to September 2020), when the COVID-19 pandemic broke out and strong COVID-19-related restrictions were in place. As the COVID-19 pandemic progressed (the second year, from September 2020 to September 2021), the values stabilized at significantly higher levels compared to the baseline measurement before COVID-19.

A worrying decline in fitness was observed in CRE, with the mean SDS values of the 6-min run dramatically declining from September 2019 to September 2020. Studies from the United States, Australia, and Europe reported that cardiorespiratory fitness was relatively stable between 1974 and 2015, with annual changes of about 0.10 SDS [42]. This makes the CRE decrease of nearly 1 SDS between September 2019 and September 2020 observed in our study even more alarming. At the final examination in September 2021, the CRE performance showed a slight improvement, but the performance level of the children still represented a significant deterioration compared with the baseline measurement in September 2019. Children who were active in a sports club showed a higher loss of CRE in the first year of observation, compared to those without sports club membership. In the second observation year (September 2020 to September 2021), children who were active in a sports club showed an increase in CRE performance, whereas children who were not active in a sports club continued to show a worsening of CRE.

Similar results relating to differences between children with and without sports club membership were observed in the SDS values for BMI. After a strong increase in both groups during the first observation period, a decrease in BMI SDS values was observed for children in sports clubs, whereas, in contrast, BMI SDS values continued to increase for children without sports club membership.

Improved performances in September 2021 compared to baseline measurements were found only in the standing long jump and action speed tests. These two unexpected results could be some kind of “training effect”. In the standing long jump, children showed a significant increase in performance during the first observation period (September 2019 to September 2020), which may be due to improved coordination of individual body parts during their jump movements. An indication for this could be that children who were not active in a sports club showed a greater increase in performance than those with sports club membership, although this took place from a significantly poorer starting level. When assessing the results of the 4 × 10 m shuttle run, it should be noted that technique plays a significant role in completing the turnarounds, and improved turning technique can lead to better overall performance. This could explain the significant increase in action speed between September 2020 and September 2021.

The other fitness tests (arm strength, flexibility, reaction speed, and whole-body coordination) performed in the pilot study [26] and in the subsequent test phases showed marginal changes, with small effects over time.

Specific fitness training does not generally take place for this age group (7 to 10 years) [43], and it therefore appears that the COVID-19-related restrictions did not have long-term negative effects on most fitness parameters. Furthermore, it was clearly observed that the restrictions in the first year (T1 to T2) had significantly more worrying effects on the fitness and health status of the children than those in the second phase of the observation period (T2 to T3). It seems that, due to the steadily increasing number of scientific reports, the restrictions were adapted to minimize negative health consequences, while still providing sufficient precautions against SARS-CoV-2 infection.

Consistent with our findings, there are already a number of studies reporting increases in BMI associated with the COVID-19 pandemic [16,19,20,23,44,45,46], but few studies [17,22] have objectively measured health-related fitness in a representative sample, and our study is the first scientific report to present results from the period prior to COVID-19, as well as from a 2-year follow-up period as the pandemic ran its course.

A major strength of our study is that all data were collected by a team of six trained sports scientists and physical educators, and that the individual measurements in the different test phases were performed by the same test director. In addition, all data collected were objectively measured directly on the participants, and we used proven and adequately reviewed fitness tests. A further strength is the large number of study participants, as it provides a general statement about the development of fitness and health status of primary school children in Austria during the COVID-19 pandemic. Another important strength is the one-year time interval between the test phases, thus excluding seasonal influences on the results.

A limitation is the lack of national reference values. An additional weakness related to the fitness reference values used is the fact that the reference values for individual fitness tests were quite old (RD) and that single results have questionable plausibility (SDS values > 2 at JS), as reported previously [26].

## 5. Conclusions

Our study shows that COVID-19-related mitigation measures had worrying negative effects on important health parameters (BMI and CRE) of Austrian primary-school-aged children during the period September 2019 to September 2020 (the first major COVID-19 lockdown), but that these dramatic effects stabilized as the COVID-19 pandemic continued. Our results indicate that, for possible future containment measures, the benefits in terms of virus transmission must be carefully weighed against the long-term negative effects on health indicated by BMI and CRE. For children without sports club membership in particular, policymakers should launch and expand initiatives and projects in schools to counteract the observed health damage (BMI and CRE) and to have a positive impact on the future development of children. In addition, easy-to-use monitoring systems [26] and educational campaigns should be implemented in the education system, as parents often have a distorted perception of their own child’s physical development [47]. A Monitoring system [26] and educational campaigns could, on the one hand, inform legal guardians about the current fitness and health status of their children and, on the other hand, inform about negative effects of undesirable developments and show possibilities and options for how to successfully counteract them.

## Figures and Tables

**Table 1:** Overall sample characteristics.

Variable	Sep-19	Sep-20	Sep-21
Age (years), Mean (SD)	7.7 (0.4)	8.7 (0.4)	9.7 (0.4)
Female sex, no. (%)	147 (48.5)		
Sports club, no. (%)	133 (43.9)		
BMI, mean (SD) in kg/m^2^	16.49 (2.41)	17.36 (2.90)	17.95 (3.23)
6MR, mean (SD) in m	906 (137)	815 (126)	860 (172)
SLJ, mean (SD) in cm	120.5 (18.1)	132.1 (19.7)	138.4 (20.1)
MB1kg, mean (SD) in cm	316.8 (66.5)	375.4 (67.3)	420.2 (74.7)
4 × 10 SHR, mean (SD) in s	15.18 (1.37)	14.97 (1.52)	13.45 (1.15)
JS, mean (SD) in no.	29.6 (6.5)	35.2 (6.8)	38.4 (6.4)
VSR, mean (SD) in cm	17.8 (8.3)	17.5 (8.7)	16.3 (8.6)
RD, mean (SD) in cm	19.3 (9.3)	19.2 (7.0)	16.4 (6.0)

Data are no. (%) or mean (SD), study population, *n* = 303; BMI = body mass index, no. = number, SD = standard deviation, 6MR = 6-min run, SLJ = standing long jump, MB1kg = medicine ball throw (1 kg), 4 × 10 SHR = 4 m × 10 m shuttle run, JS = jumping sideways, VSR = V sit-and-reach test, RD = ruler drop test, kg = kilogram, m = meter, cm = centimeter, s = second.

**Table 2:** Standard deviation scores (SDSs) and traditional z-scores (z-values) according to international reference values.

				∆ Sep-19 to Sep-20		∆ Sep-20 to Sep-21	
Variable	Sep-19	Sep-20	Sep-21	Mean ∆	95% CI	Mean ∆	95% CI
BMI SDS, mean (SD)	0.32 (1.02)	0.49 (1.04)	0.48 (1.04)	0.17 (0.38)	0.12 to 0.21	−0.01 (0.34)	−0.04 to 0.04
6MR SDS, mean (SD)	0.49 (1.12)	−0.43 (0.93)	−0.38 (1.21)	−0.92 (0.95)	−1.02 to −0.81	0.05 (1.12)	−0.08 to 0.18
SLJ SDS, mean (SD)	0.11 (1.00)	0.47 (1.07)	0.29 (1.02)	0.36 (0.90)	0.26 to 0.46	−0.18 (0.74)	−0.26 to −0.10
MB1kg z-value, mean (SD)	0.09 (1.00)	0.09 (0.93)	−0.12 (0.92)	0.00 (0.89)	−0.10 to 0.10	−0.21 (0.75)	−0.30 to −0.13
4 × 10 SHR SDS, mean (SD)	−0.31 (0.85)	−0.64 (0.96)	0.02 (0.93)	−0.33 (0.88)	−0.42 to −0.22	0.66 (0.86)	0.56 to 0.76
JS z-value, mean (SD)	2.24 (1.40)	2.37 (1.26)	2.18 (1.06)	0.14 (1.27)	−0.01 to 0.28	−0.19 (0.98)	−0.30 to −0.08
VSR SDS, mean (SD)	0.35 (1.43)	0.36 (1.46)	0.19 (1.42)	0.01 (0.85)	−0.09 to 0.10	−0.17 (0.86)	−0.27 to −0.07
RD z-value, mean (SD)	1.09 (1.28)	0.81 (1.10)	1.07 (1.06)	−0.28 (1.49)	−0.45 to −0.11	0.26 (1.31)	0.11 to 0.40

Data are mean (SD); sample size for study population, *n* = 303; Sep-19 = September 2019, Sep-20 = September 2020, and Sep-21 = September 2021; ∆ = change over time, CI = confidence interval, BMI = body mass index, SD = standard deviation, 6MR = 6-min run, SLJ = standing long jump, MB1kg = medicine ball throw (1 kg), 4 × 10 SHR = 4 m × 10 m shuttle run, JS = jumping sideways, VSR = V sit-and-reach test, RD = ruler drop test, m = meter, SDS = standard deviation score, z-value = traditional z-score standardization.

**Table 3:** Mixed ANOVAs for anthropometrics and fitness.

Variable	Effects	Time and Subgroups	df	F	*p*-Value	η_p_^2^	Power ^a^
BMI SDS	Between-subjects effects	Sex	1	1.946	0.164	0.006	0.285
		Sports Club	1	0.892	0.346	0.003	0.156
		Sex × Sports Club	1	1.260	0.263	0.004	0.201
		Error	299				
	Within-subjects effects	Time (T1, T2, and T3)	1.895	33.759	<0.001	0.101	1.000
		Time × Sex	1.895	6.258	0.002	0.021	0.882
		Time × Sports Club	1.895	5.571	0.005	0.018	0.841
		Time × Sex × Sports Club	1.895	3.139	0.047	0.010	0.586
		Error (Time)	566.515				
6MR SDS	Between-subjects effects	Sex	1	6.272	0.013	0.021	0.704
		Sports Club	1	16.129	<0.001	0.051	0.979
		Sex × Sports Club	1	0.989	0.321	0.003	0.168
		Error	299				
	Within-subjects effects	Time (T1, T2, and T3)	1.903	118.204	<0.001	0.283	1.000
		Time × Sex	1.903	1.487	0.228	0.005	0.310
		Time × Sports Club	1.903	0.557	0.565	0.002	0.140
		Time × Sex × Sports Club	1.903	0.821	0.435	0.003	0.187
		Error (Time)	568.960				
SLJ SDS	Between-subjects effects	Sex	1	1.033	0.310	0.003	0.173
		Sports Club	1	4.052	0.045	0.013	0.519
		Sex × Sports Club	1	1.004	0.317	0.003	0.170
		Error	299				
	Within-subjects effects	Time (T1, T2, andT3)	1.904	24.110	<0.001	0.075	1.000
		Time × Sex	1.904	1.873	0.157	0.006	0.380
		Time × Sports Club	1.904	0.109	0.888	0.000	0.066
		Time × Sex × Sports Club	1.904	1.204	0.300	0.004	0.257
		Error (Time)	569.303				
MB1kg z-value	Between-subjects effects	Sex	1	2.564	0.110	0.009	0.358
		Sports Club	1	4.528	0.034	0.015	0.564
		Sex × Sports Club	1	5.310	0.022	0.017	0.632
		Error	299				
	Within-subjects effects	Time (T1, T2, and T3)	1.922	8.229	<0.001	0.027	0.955
		Time × Sex	1.922	2.633	0.075	0.009	0.513
		Time × Sports Club	1.922	0.934	0.391	0.003	0.208
		Time × Sex × Sports Club	1.922	2.060	0.130	0.007	0.416
		Error (Time)	574.679				
4 × 10 SHR SDS	Between-subjects effects	Sex	1	2.552	0.111	0.008	0.357
		Sports Club	1	13.197	<0.001	0.042	0.952
		Sex × Sports Club	1	0.004	0.953	0.000	0.050
		Error	299				
	Within-subjects effects	Time (T1, T2, and T3)	2	77.420	<0.001	0.206	1.000
		Time × Sex	2	1.013	0.364	0.003	0.227
		Time × Sports Club	2	1.716	0.181	0.006	0.361
		Time × Sex × Sports Club	2	1.181	0.308	0.004	0.259
		Error (Time)	598				
JS z-value	Between-subjects effects	Sex	1	7.805	0.006	0.025	0.795
		Sports Club	1	8.923	0.003	0.029	0.846
		Sex × Sports Club	1	4.586	0.033	0.015	0.569
		Error	299				
	Within-subjects effects	Time (T1, T2, and T3)	1.828	5.107	0.008	0.017	0.796
		Time × Sex	1.828	1.625	0.200	0.005	0.328
		Time × Sports Club	1.828	2.411	0.096	0.008	0.464
		Time × Sex × Sports Club	1.828	0.149	0.843	0.000	0.072
		Error (Time)	546.628				
VSR SDS	Between-subjects effects	Sex	1	25.714	<0.001	0.079	0.999
		Sports Club	1	2.871	0.091	0.010	0.393
		Sex × Sports Club	1	0.108	0.743	0.000	0.062
		Error	299				
	Within-subjects effects	Time (T1, T2, and T3)	2	8.343	<0.001	0.027	0.963
		Time × Sex	2	3.063	0.047	0.010	0.591
		Time × Sports Club	2	0.587	0.557	0.002	0.148
		Time × Sex × Sports Club	2	0.256	0.774	0.001	0.090
		Error (Time)	598				
RD z-value	Between-subjects effects	Sex	1	5.010	0.026	0.016	0.607
		Sports Club	1	2.088	0.149	0.007	0.302
		Sex × Sports Club	1	0.368	0.545	0.001	0.093
		Error	299				
	Within-subjects effects	Time (T1, T2, and T3)	1.941	5.525	0.005	0.018	0.844
		Time × Sex	1.941	0.313	0.725	0.001	0.099
		Time × Sports Club	1.941	1.604	0.203	0.005	0.335
		Time × Sex × Sports Club	1.941	0.642	0.522	0.002	0.156
		Error (Time)	580.445				

^a^ Observed power computed by using alpha = 0.05. ANOVA = analysis of variance, BMI = body mass index, df = degrees of freedom, F = test statistic, η_p_^2^ = partial eta square, IOTF = BMI SDS based on International Obesity Taskforce reference centile curves [38], SD = standard deviation; SDS = standard deviation score, z-value = traditional z-score standardization; 6MR = 6-min run, SLJ = standing long jump, MB1kg = medicine ball throw (1 kg), 4 × 10 SHR = 4 m × 10 m shuttle run, JS = jumping sideways, VSR = V sit-and-reach test, RD = ruler drop test, m = meter, SDS = standard deviation score, z-value = traditional z-score standardization; T1 = baseline measurements in September 2019, T2 = follow-up measurements in September 2020, T3 = follow-up measurements in September 2021.

## Data Availability

The data presented in this study are available upon request from the corresponding author. The data are not publicly available due to privacy/ethical restriction.

## Supplementary Materials

The following are available online at https://www.mdpi.com/article/10.3390/sports10050078/s1. Table S1: Restriction levels for children in Austria and detailed description of the restrictions for Austrian children in primary school from 31 January 2020 to 30 September 2021 in relation to the OxCGRT Stringency. Table S2: Overall sample characteristics study population vs. loss at follow-up. Table S3: Additional sample characteristics for subgroups sex and sports club membership. Table S4: Standard deviation scores (SDSs) and traditional z-scores (z-values) according to international reference values for subgroups sex and sports club membership. Table S5: Post hoc tests for anthropometrics and fitness for the main effect time and interactions for time * sex and time * sports club membership based on the estimated marginal means. Figure S1: OxCGRT Stringency Index. Figure 1 shows the Oxford COVID-19 Government Response Tracker (OxCGRT) Stringency Index for Austria between 31 January 2020 and 30 September 2021. Figure S2: Restriction levels for primary school children. The detailed description of this classification method used in Figure S2 is available in Table S1.
