# Supplementary material for: Change in BMI and Fitness among Primary School Children in Austria: A 24-Month Follow-Up Study of 303 Children Measured before and during the Ongoing COVID-19 Pandemic
Source: Sports (Basel). 2022 May 19;10(5):78. doi: 10.3390/sports10050078 (PMC9147516; doi:10.3390/sports10050078)
Supplement: Supplementary file 1 [file sports-10-00078-s001.zip › sports-1717701-supplementary.pdf]

Change in BMI and Fitness among Primary School Children in Austria: A 24-Month Follow-Up Study of 303 Children Measured before and during the Ongoing COVID-19 Pandemic

Supplementary material

Gerald Jarnig, Reinhold Kerbl and Mireille N.M. van Poppel

This supplementary material has been provided by the authors to give readers additional information about their work.

Table S1. Restriction levels for children in Austria and detailed description of the restrictions for Austrian children in primary school from January 31, 2020 to September 30, 2021 in relation to the OxCGRT Stringency.

Table S2. Overall sample characteristics study population vs. loss at follow-up.

Table S3. Additional sample characteristics for subgroups sex and sports club membership.

Table S4. Standard deviation scores (SDS) and traditional z-scores (z-values) according to international reference values for subgroups sex and sports club membership.

Table S5. Post hoc tests for anthropometrics and fitness for the main effect time and interactions for time*sex and time*sports club membership based on the estimated marginal means.

Figure S1. OxCGRT Stringency Index; Figure S1 shows the Oxford COVID-19 Government Response Tracker (OxCGRT) Stringency Index for Austria between January 31, 2020 and September 30, 2021.

Figure S2. Restriction levels for primary school children; The detailed description of this classification method used in Figure S2 is available in Table S1.

Table S1. Restriction levels for children in Austria and detailed description of the restrictions for Austrian children in primary school from January 31, 2020 to September 30, 2021 in relation to the OxCGRT Stringency.

Period	Index[a]	school structure		Physical education in school		Sports and leisure facilities		Sport at the club		Stay at home requirements		Sum of restrictions in the school
01-31-2020 – 02-23-2020	0.00	A	0	A	0	A	0	A	0	A	0	0
02-24-2020 – 03-08-2020	11.11	A	0	A	0	A	0	A	0	A	0	0
03-09-2020 – 03-11-2020	19.44	A	0	A	0	A	0	A	0	A	0	0
03-12-2020	34.26	A	0	A	0	A	0	A	0	A	0	0
03-13-2020 – 03-15-2020	48.15	A	0	A	0	A	0	A	0	A	0	0
03-16-2020 – 04-13-2020	81.48	B	3	D	3	E	3	G	3	I	3	15
04-14-2020 – 04-22-2020	77.78	B	3	D	3	E	3	G	3	I	3	15
04-23-2020 – 04-30-2020	75.00	B	3	D	3	E	3	G	3	I	3	15
05-01-2020	67.59	B	3	D	3	F	2	G	3	J	2	13
05-02-2020 – 05-10-2020	64.81	B	3	D	3	F	2	G	3	J	2	13
05-11-2020 – 05-17-2020	59.26	B	3	D	3	F	2	G	3	J	2	13
05-18-2020 – 05-28-2020	59.26	C	2	D	3	F	2	G	3	J	2	12
05-29-2020 – 06-02-2020	53.70	C	2	D	3	F	2	H	2	J	2	11
06-03-2020	50.00	C	2	D	3	F	2	H	2	J	2	11
06-04-2020 – 07-01-2020	47.22	C	2	D	3	F	2	H	2	J	2	11
07-02-2020 – 07-09-2020	47.22	C	2	D	3	A	0	L	1	A	0	6
07-10-2020 – 09-05-2020	35.19	K	0	K	0	A	0	L	1	A	0	1
09-06-2020 – 09-13-2020	36.11	K	0	K	0	A	0	L	1	A	0	1
09-14-2020 – 09-16-2020	36.11	M	1	A	0	A	0	L	1	J	1	3
09-17-2020 – 09-28-2020	37.04	M	1	A	0	A	0	L	1	J	1	3
09-29-2020 – 10-12-2020	40.74	M	1	A	0	A	0	L	1	J	1	3
10-13-2020 – 10-16-2020	44.91	M	1	A	0	A	0	L	1	O	2	4
10-17-2020 – 10-22-2020	58.80	M	1	A	0	A	0	L	1	O	2	4
10-23-2020	60.19	M	1	A	0	A	0	L	1	O	2	4
10-24-2020 – 10-26-2020	60.19	K	0	K	0	F	1	G	3	O	2	6
10-27-2020 – 11-01-2020	64.81	K	0	K	0	F	1	G	3	O	2	6
11-02-2020	75.00	K	0	K	0	F	1	G	3	O	2	6
11-03-2020 – 11-16-2020	75.00	M	0	N	2	F	1	G	3	O	2	8
11-17-2020 – 12-06-2020	82.41	B	3	D	3	F	1	G	3	O	2	12
12-07-2020 – 12-23-2020	71.30	M	1	N	2	F	1	G	3	J	1	8
12-24-2020 – 12-25-2020	82.41	K	3	K	3	F	1	G	3	O	2	12
12-26-2020 – 01-06-2021	82.41	K	3	K	3	F	1	G	3	O	2	12
01-07-2021 – 01-18-2021	82.41	B	3	D	3	F	1	G	3	O	2	12
01-19-2021 – 02-05-2021	82.41	C	2	N	2	F	1	G	3	J	1	9
02-06-2021 – 02-07-2021	82.41	K	3	K	3	F	1	G	3	J	1	11
02-08-2021 – 02-11-2021	74.07	K	3	K	3	F	1	G	3	J	1	11
02-12-2021 – 02-14-2021	79.63	K	3	K	3	F	1	G	3	J	1	11
02-15-2021 – 03-14-2021	75.93	M	1	N	2	F	1	G	3	J	1	8
03-15-2021 – 03-31-2021	73.15	M	1	N	2	F	1	H	2	J	1	7
04-01-2021 – 04-05-2021	75.00	M	1	N	2	F	1	H	2	J	1	7
04-06-2021 – 04-25-2021	78.70	M	1	N	1	F	1	H	2	J	1	6
04-26-2021 – 05-02-2021	76.85	M	1	N	1	F	1	H	2	J	1	6
05-02-2021 – 05-10-2021	75.00	M	1	N	1	F	1	H	2	J	1	6
05-11-2021 – 05-15-2021	73.15	M	1	N	1	F	1	H	2	J	1	6
05-16-2021 – 05-18-2021	65.74	M	1	N	1	F	1	H	2	J	1	6
05-19-2021 – 06-09-2021	68.52	M	1	P	1	A	0	Q	1	A	0	3
06-10-2021 – 06-30-2021	60.19	M	1	P	1	A	0	Q	1	A	0	3

07-01-2021 – 08-10-2021	49.07	A	0	A	0	A	0	A	0	A	0	0
08-11-2021 – 08-24-2021	56.02	A	0	A	0	A	0	A	0	A	0	0
08-25-2021 – 09-08-2021	58.80	A	0	A	0	A	0	A	0	A	0	0
09-09-2021 – 09-12-2021	49.07	A	0	A	0	A	0	A	0	A	0	0
09-13-2021 – 09-14-2021	49.07	A	0	R	1	A	0	A	0	A	0	1
09-15-2021 – 09-17-2021	51.85	A	0	R	1	A	0	S	1	A	0	2
09-18-2021 – 09-28-2021	60.19	A	0	R	1	A	0	S	1	A	0	2
09-29-2021 – 09-30-2021	51.85	A	0	R	1	A	0	S	1	A	0	2

a = COVID-19: Stringency Index der Regierung, 0 = no restrictions, 1 = low restrictions, 2 = medium restrictions, 3 = high restrictions; A = no restrictions, B = Distance learning in primary school, C = Lessons in primary school in shifts / home schooling are possible and recommended, D = no physical education in school, E = Closures of all public playgrounds and sports facilities, F = public playgrounds and sports grounds are reopened, minimum distance must be maintained, G = Club sport is forbidden, H = Club sport is possible under severe restrictions and conditions, I = Required with exceptions, J = Restricted freedom of movement / keeping a minimum distance; K = holidays, L = Practice of club sports is allowed under submission of prevention concepts, M = ordinary lessons in elementary school / home schooling possible, N = Limited sports activities in elementary school, O = Recommended to stay at home, P = ordinary (outdoor) physical education in school, Q = Practice of club sports is allowed under submitting prevention concepts, R = restrictions in school sports possible, S = Participation in club sports is possible with presentation of COVID-19 vaccination, test and recovery certificates / COVID-19 prevention concept required.

Table S2. Overall sample characteristics study population vs. loss at follow-up.

Variable	Study population	loss at follow-up	X^2	t	p-lvl	*p*-Value
Age (years)	7.7 (0.4)	7.8 (0.5)		1.756		0.08
Female Sex	147 (48.5%)	57 (50.9%)	0.185			0.67
Sports Club membership	133 (43.9%)	45 (40.2%)	0.461			0.50
BMI SDS_{IOTF}	0.32 (1.02)	0.32 (1.24)		0.010		0.99
6MR SDS (DüMo)	0.49 (1.12)	0.12 (1.01)		3.072	**	0.002
SLJ SDS (DüMo)	0.10 (1.00)	-0.15 (1.02)		2.322	*	0.021
MB1kg z-value (KATS-K)	0.09 (1.00)	0.04 (1.03)		0.420		0.67
4 × 10 SHR SDS (MCA)	-0.31 (0.85)	-0.72 (0.93)		4.245	***	<.001
JS z-value (MoMo)	2.24 (1.40)	2.45 (1.57)		1.4		0.16
VSR SDS (MAKFIT)	0.35 (1.43)	0.28 (1.40)		0.482		0.63
RD z-value (F&K)	1.09 (1.28)	1.46 (0.89)		3.353	**	0.001

Data are *n* (%) or mean (SD); sample size for study population, $n = 303$; for loss at follow up, $n = 112$, X^2 = chi-square test value; t = test statistic *t*-test, p-lvl (*p*-value level) * = $p < 0.05$, ** = $p < 0.01$, *** = $p < 0.001$; BMI = body mass index, SD = standard deviation, 6MR = 6 minute run, SLJ = standing long jump, MB1kg = medicine ball throw (1 kilogram), 4 x 10 SHR = 4-m x 10-m shuttle run, JS = jumping sideways, VSR = V sit-and-reach Test, RD = ruler drop test, m = meter, SDS = standard deviation score, z-value = traditional z-score standardization; DüMo = Düsseldorfer Modell [1], KATS-K = Karlsruher test system [2], MCA = Motor Competence Assessment [3], MoMo = Motor Module [4], MAKFIT = Macedonian fitness meter [5], F&K = Sport motor tests by Fetz and Kornexl [6].

Table S3. Additional sample characteristics for subgroups sex and sports club membership.

		Sep-19	Sep-20	Sep-21
**BMI, mean (SD)**	Girls	16.83 (2.78)	17.67 (3.33)	18.35 (3.69)
	Boys	16.16 (1.97)	17.06 (2.39)	17.58 (2.68)
	Sports Club	16.22 (1.81)	17.01 (2.24)	17.42 (2.47)
	No Sports Club	16.69 (2.79)	17.63 (3.30)	18.37 (3.67)
**6MR, mean (SD) in m**	Girls	843 (117)	769 (109)	790 (147)
	Boys	965 (129)	857 (126)	926 (167)
	Sports Club	956 (131)	855 (128)	920 (174)
	No Sports Club	866 (129)	783 (114)	813 (154)
**SLJ, mean (SD) in cm**	Girls	114.0 (17.0)	126.5 (19.4)	132.9 (19.3)
	Boys	126.5 (17.0)	137.4 (18.5)	143.6 (19.6)
	Sports Club	124.9 (17.4)	135.9 (19.6)	143.3 (19.7)
	No Sports Club	117.0 (17.9)	129.2 (19.3)	134.6 (19.7)
**MB1kg, mean (SD) in cm**	Girls	285.8 (52.7)	345.5 (58.3)	387.7 (64.0)
	Boys	346.1 (64.9)	403.5 (63.0)	450.9 (71.1)
	Sports Club	337.6 (67.8)	392.1 (72.9)	439.5 (73.3)
	No Sports Club	300.6 (60.8)	362.1 (72.9)	405.2 (72.5)
**4x10SHR, mean (SD) in sec.**	Girls	15.62 (1.44)	15.29 (1.42)	13.89 (1.17)
	Boys	14.76 (1.16)	14.67 (1.56)	13.04 (0.98)
	Sports Club	14.84 (1.14)	14.55 (1.40)	13.05 (1.00)
	No Sports Club	15.44 (1.48)	15.30 (1.53)	13.77 (1.16)
**JS, mean (SD) in No.**	Girls	28.9 (5.9)	33.8 (6.0)	36.9 (5.6)
	Boys	30.2 (7.0)	36.4 (7.2)	39.9 (6.8)
	Sports Club	30.6 (6.6)	37.3 (6.8)	39.9 (6.5)
	No Sports Club	28.8 (6.3)	33.5 (6.3)	37.3 (6.0)
**VSR, mean (SD) in cm**	Girls	20.8 (8.4)	20.5 (8.3)	19.0 (8.8)
	Boys	15.1 (7.1)	14.7 (8.1)	13.7 (7.6)
	Sports Club	17.6 (8.0)	17.3 (8.7)	15.9 (8.6)
	No Sports Club	18.0 (8.5)	17.7 (8.7)	16.6 (8.7)
**RD, mean (SD) in cm**	Girls	20.2 (9.1)	20.0 (7.3)	17.0 (6.2)
	Boys	18.3 (9.4)	18.3 (6.6)	15.8 (6.6)
	Sports Club	19.2 (9.3)	18.1 (5.9)	15.5 (6.0)
	No Sports Club	19.3 (9.3)	20.0 (7.6)	17.1 (6.0)

Data are No (%) or mean (SD); study population, $n = 303$; BMI = body mass index, No. = number, SD = standard deviation, 6MR = 6-minute run, SLJ = standing long jump, MB1kg = medicine ball throw (1 kilogram), 4 x 10 SHR = 4-m x 10-m shuttle run, JS = jumping sideways, VSR = V sit-and-reach Test, RD = ruler drop test, m = meter, cm = centimeter, sec. = seconds.

Table S4. Standard deviation scores (SDS) and traditional z-scores (z-values) according to international reference values for subgroups sex and sports club membership.

Variable		Sep-19	Sep-20	Sep-21	Δ Sep-19 to Sep-20	95% CI – Δ Sep-19 to Sep-20	Δ Sep-20 to Sep-21	95% CI – Δ Sep-20 to Sep-21
SDS BMIIOTF, mean (SD)	Girls	0.48 (1.08)	0.58 (1.13)	0.59 (1.10)	0.10 (0.37)	0.04 to 0.16	0.01 (0.31)	-0.04 to 0.06
	Boys	0.17 (0.95)	0.40 (0.95)	0.39 (0.96)	0.23 (0.37)	0.17 to 0.28	-0.01 (0.38)	-0.07 to 0.05
	Sports Club	0.25 (0.89)	0.40 (0.91)	0.35 (0.92)	0.15 (0.36)	0.09 to 0.21	-0.05 (0.33)	- 0.11 to 0.00
	No Sports Club	0.37 (1.11)	0.55 (1.14)	0.59 (1.11)	0.18 (0.39)	0.12 to 0.23	0.04 (0.35)	-0.01 to 0.09
6MR SDS (DüMo), mean (SD)	Girls	0.28 (1.10)	-0.55 (0.91)	-0.65 (1.16)	-0.83 (0.95)	-0.98 to -0.67	-0.10 (1.01)	-0.27 to 0.06
	Boys	0.69 (1.11)	-0.31 (0.93)	-0.12 (1.20)	-1.00 (0.94)	-1.15 to -0.85	0.19 (1.20)	0.00 to 0.38
	Sports Club	0.79 (1.07)	-0.20 (0.93)	-0.03 (1.21)	-0.99 (0.89)	-1.14 to -0.83	0.17 (1.29)	-0.06 to 0.39
	No Sports Club	0.25 (1.10)	-0.61 (0.88)	-0.65 (1.14)	-0.86 (0.98)	-1.01 to -0.71	-0.04 (0.96)	-0.18 to 0.10
SLJ SDS (DüMo), mean (SD)	Girls	-0.05 (0.99)	0.40 (1.12)	0.24 (1.02)	0.44 (0.97)	0.29 to 0.60	-0.16 (0.71)	-0.28 to -0.04
	Boys	0.25 (0.99)	0.53 (1.02)	0.33 (1.02)	0.28 (0.83)	0.15 to 0.42	-0.20 (0.77)	-0.32 to -0.08
	Sports Club	0.27 (0.98)	0.60 (1.05)	0.44 (1.00)	0.33 (0.82)	0.19 to 0.47	-0.15 (0.69)	-0.27 to -0.04
	No Sports Club	-0.03 (0.99)	0.36 (1.07)	0.16 (1.02)	0.39 (0.97)	0.24 to 0.54	-0.20 (0.78)	-0.32 to -0.08
MB1kg z-value (KATS-K), mean (SD)	Girls	0.07 (1.01)	0.20 (0.93)	-0.02 (0.91)	0.13 (0.96)	-0.03 to 0.29	-0.22 (0.80)	-0.35 to -0.09
	Boys	0.11 (1.00)	-0.01 (0.92)	-0.21 (0.92)	-0.12 (0.80)	-0.24 to 0.01	-0.20 (0.70)	-0.31 to -0.09
	Sports Club	0.26 (0.96)	0.15 (0.92)	-0.05 (0.89)	-0.11 (0.85)	-0.26 to 0.03	-0.20 (0.65)	-0.31 to -0.09
	No Sports Club	-0.04 (1.02)	0.05 (0.93)	-0.18 (0.94)	0.09 (0.91)	-0.05 to 0.23	-0.23 (0.82)	-0.35 to -0.10
4x10SHR SDS (MCA)	Girls	-0.43 (0.93)	-0.74 (0.96)	-0.16 (0.94)	-0.30 (0.92)	-0.45 to -0.15	0.57 (0.88)	0.43 to 0.72
	Boys	-0.20 (0.77)	-0.54 (0.96)	0.19 (0.89)	-0.35 (0.86)	-0.48 to -0.21	0.74 (0.83)	0.61 to 0.87

, mean (SD)	Sports Club	-0.16 (0.76)	-0.41 (0.89)	0.30 (0.88)	-0.25 (0.88)	- 0.40 to - 0.10	0.71 (0.89)	0.55 to 0.86
	No Sports Club	-0.43 (0.90)	-0.81 (0.98)	-0.19 (0.91)	-0.38 (0.89)	-0.52 to -0.25	0.62 (0.83)	0.49 to 0.75
JS z-value (GMT), mean (SD)	Girls	2.08 (1.31)	2.12 (1.18)	1.91 (0.95)	0.04 (1.22)	-0.16 to 0.24	-0.21 (0.91)	-0.36 to -0.07
	Boys	2.38 (1.46)	2.61 (1.29)	2.44 (1.10)	0.22 (1.32)	0.02 to 0.43	-0.17 (1.03)	-0.33 to -0.01
	Sports Club	2.47 (1.39)	2.76 (1.22)	2.43 (1.06)	0.28 (1.20)	0.08 to 0.49	-0.32 (0.88)	-0.48 to -0.18
	No Sports Club	2.05 (1.37)	2.07 (1.21)	1.99 (1.02)	0.02 (1.33)	-0.18 to 0.22	-0.08 (1.03)	-0.24 to 0.07
VSR SDS (MAKFIT), mean (SD)	Girls	0.77 (1.48)	0.76 (1.42)	0.50 (1.48)	-0.01 (0.92)	-0.16 to 0.14	-0.26 (0.88)	-0.40 to -0.12
	Boys	-0.04 (1.26)	-0.02 (1.40)	-0.11 (1.30)	0.02 (0.78)	-0.10 to 0.14	-0.09 (0.83)	-0.22 to 0.04
	Sports Club	0.35 (1.38)	0.36 (1.47)	0.18 (1.42)	0.01 (0.77)	-0.13 to 0.14	-0.18 (0.85)	-0.33 to -0.04
	No Sports Club	0.35 (1.47)	0.36 (1.45)	0.19 (1.43)	0.01 (0.91)	-0.13 to 0.14	-0.16 (0.87)	-0.30 to -0.03
RD z-value (Fetz), mean (SD)	Girls	0.96 (1.24)	0.69 (1.14)	0.89 (0.89)	-0.27 (1.51)	-0.51 to -0.02	0.20 (1.14)	0.02 to 0.39
	Boys	1.21 (1.31)	0.92 (1.06)	1.23 (1.17)	-0.29 (1.48)	-0.52 to -0.05	0.31 (1.45)	0.08 to 0.53
	Sports Club	1.10 (1.26)	0.98 (0.95)	1.23 (1.13)	-0.12 (1.40)	-0.36 to 0.12	0.25 (1.29)	0.03 to 0.47
	No Sports Club	1.07 (1.30)	0.67 (1.19)	0.93 (0.98)	-0.40 (1.55)	-0.63 to -0.16	0.26 (1.32)	0.06 to 0.46

Data are mean (SD); sample size for study population, n = 303; Sep-19 = September 2019, Sep-20 = September 2020, Sep-21 = September 2021, Δ = change over time, CI = confidence interval, BMI = body mass index, SD = standard deviation, 6MR = 6 minute run, SLJ = standing long jump, MB1kg = medicine ball throw (1 kilogram), 4 x 10 SHR = 4-m x 10-m shuttle run, JS = jumping sideways, VSR = V sit-and-reach Test, RD = ruler drop test, m = meter, SDS = standard deviation score, z-value = traditional z-score standardization; DüMo = Düsseldorfer Modell [1], KATS-K = Karlsruher test system [2], MCA = Motor Competence Assessment [3], MoMo = Motor Module [4], MAKFIT = Macedonian fitness meter [5], F&K = Sport motor tests by Fetz and Kornexl [6].

Table S5. Post hoc tests for anthropometrics and fitness for the main effect time and interactions for time*sex and time*sports club membership based on the estimated marginal means.

Variable	Subgroups	Pairwise comparisons	Mean diff (95% CI)	SE	p-lvl	*P* Value[a]
SDS BMI$_{IOTF}$	Time	T1 to T2	-0,162* (-0.218 to -0.107)	0.023	***	<.001
		T1 to T3	-0,170* (-0.232 to -0.108)	0.026	***	<.001
		T2 to T3	-0.008 (-0.059 to 0.043)	0.021		>.99
	Time*Sex (Girls)	T1 to T2	-0,091* (-0.175 to -0.008)	0.035	*	0.027
		T1 to T3	-0,098* (-0.191 to -0.004)	0.039	*	0.037
		T2 to T3	-0.006 (-0.083 to 0.070)	0.032		>.99
	Time*Sex (Boys)	T1 to T2	-0,233* (-0.306 to -0.160)	0.030	***	<.001
		T1 to T3	-0,242* (-0.324 to -0.161)	0.034	***	<.001
		T2 to T3	-0.009 (-0.076 to 0.057)	0.028		>.99
	Time*Sports Club (Yes)	T1 to T2	-0,128* (-0.213 to -0.042)	0.035	**	0.001
		T1 to T3	-0.092 (-0.187 to 0.003)	0.040		0.061
		T2 to T3	0.035 (-0.042 to 0.113)	0.032		0.822
	Time*Sports Club (No)	T1 to T2	-0,197* (-0.268 to -0.126)	0.030	***	<.001
		T1 to T3	-0,248* (-0.328 to -0.168)	0.033	***	<.001
		T2 to T3	-0.051 (-0.116 to 0.014)	0.027		0.177
6MR SDS (DüMo)	Time	T1 to T2	0,924* (0.783 to 1.065)	0.059	***	<.001
		T1 to T3	0,843* (0.671 to 1.014)	0.071	***	<.001
		T2 to T3	-0.081 (-0.248 to 0.085)	0.069		0.716
	Time*Sex (Girls)	T1 to T2	0,854* (0.641 to 1.067)	0.088	***	<.001
		T1 to T3	0,886* (0.628 to 1.145)	0.107	***	<.001
		T2 to T3	0.032 (-0.218 to 0.282)	0.104		>.99
	Time*Sex (Boys)	T1 to T2	0,994* (0.808 to 1.181)	0.077	***	<.001
		T1 to T3	0,799* (0.573 to 1.025)	0.094	***	<.001
		T2 to T3	-0.195 (-0.414 to 0.024)	0.091		0.098
	Time*Sports Club (Yes)	T1 to T2	0,965* (0.748 to 1.182)	0.090	***	<.001
		T1 to T3	0,814* (0.550 to 1.077)	0.109	***	<.001
		T2 to T3	-0.151 (-0.406 to 0.103)	0.106		0.461
	Time*Sports Club (No)	T1 to T2	0,883* (0.702 to 1.065)	0.075	***	<.001
		T1 to T3	0,872* (0.652 to 1.092)	0.091	***	<.001
		T2 to T3	-0.012 (-0.225 to 0.202)	0.089		>.99
SLJ SDS (DüMo)	Time	T1 to T2	-0,362* (-0.498 to -0.227)	0.056	***	<.001
		T1 to T3	-0,164* (-0.294 to -0.034)	0.054	**	0.008
		T2 to T3	0,199* (0.087 to 0.310)	0.046	***	<.001
	Time*Sex (Girls)	T1 to T2	-0,440* (-0.643 to -0.237)	0.084	***	<.001
		T1 to T3	-0,259* (-0.454 to -0.063)	0.081	**	0.005
		T2 to T3	0,181* (0.014 to 0.349)	0.070	*	0.029
	Time*Sex (Boys)	T1 to T2	-0,285* (-0.463 to -0.107)	0.074	***	<.001
		T1 to T3	-0.069 (-0.240 to 0.103)	0.071		>.99

		T2 to T3	0,216* (0.070 to 0.363)	0.061	**	0.001
	Time*Sports Club (Yes)	T1 to T2	-0,357* (-0.564 to -0.150)	0.086	***	<.001
		T1 to T3	-0.182 (-0.381 to 0.018)	0.083		0.087
		T2 to T3	0,175* (0.005 to 0.346)	0.071	*	0.042
	Time*Sports Club (No)	T1 to T2	-0,368* (-0.541 to -0.194)	0.072	***	<.001
		T1 to T3	-0.146 (-0.312 to 0.021)	0.069		0.109
		T2 to T3	0,222* (0.079 to 0.635)	0.059	**	0.001
MB1kg z-value (KATS-K)	Time	T1 to T2	0.009 (-0.123 to 0.141)	0.055		>.99
		T1 to T3	0,186* (0.057 to 0.316)	0.054	**	0.002
		T2 to T3	0,177* (0.066 to 0.289)	0.046	***	<.001
	Time*Sex (Girls)	T1 to T2	-0.087 (-0.286 to 0.111)	0.083		0.874
		T1 to T3	0.078 (-0.117 to 0.273)	0.081		>.99
		T2 to T3	0.165 (-0.002 to 0.333)	0.070		0.055
	Time*Sex (Boys)	T1 to T2	0.106 (-0.068 to 0.280)	0.072		0.434
		T1 to T3	0,295* (0.124 to 0.465)	0.071	***	<.001
		T2 to T3	0,189* (0.042 to 0.336)	0.061	**	0.006
	Time*Sports Club (Yes)	T1 to T2	0.079 (-0.124 to 0.282)	0.084		>.99
		T1 to T3	0,231* (0.032 to 0.430)	0.083	*	0.016
		T2 to T3	0.152 (-0.019 to 0.323)	0.071		0.100
	Time*Sports Club (No)	T1 to T2	-0.061 (-0.230 to 0.109)	0.070		>.99
		T1 to T3	0.142 (-0.024 to 0.308)	0.069		0.122
		T2 to T3	0,203* (0.059 to 0.346)	0.059	**	0.002
4x10SHR SDS (MCA)	Time	T1 to T2	0,337* (0.205 to 0.469)	0.055	***	<.001
		T1 to T3	-0,322* (-0.444 to -0.200)	0.051	***	<.001
		T2 to T3	-0,659* (-0.787 to -0.530)	0.053	***	<.001
	Time*Sex (Girls)	T1 to T2	0,299* (0.100 to 0.497)	0.082	**	0.001
		T1 to T3	-0,285* (-0.468 to -0.101)	0.076	**	0.001
		T2 to T3	-0,584* (-0.777 to -0.390)	0.080	***	<.001
	Time*Sex (Boys)	T1 to T2	0,375* (0.201 to 0.549)	0.072	***	<.001
		T1 to T3	-0,359* (-0.520 to -0.199)	0.067	***	<.001
		T2 to T3	-0,734* (-0.904 to -0.565)	0.070	***	<.001
	Time*Sports Club (Yes)	T1 to T2	0,263* (0.060 to 0.465)	0.084	**	0.006
		T1 to T3	-0,414* (-0.601 to -0.228)	0.078	***	<.001
		T2 to T3	-0,677* (-0.874 to -0.480)	0.082	***	<.001
	Time*Sports Club (No)	T1 to T2	0,411* (0.242 to 0.581)	0.070	***	<.001
		T1 to T3	-0,229* (-0.386 to -0.073)	0.065	**	0.001
		T2 to T3	-0,641* (-0.806 to -0.476)	0.069	***	<.001
JS z-value (GMT)	Time	T1 to T2	-0.152 (-0.342 to 0.039)	0.079		0.168
		T1 to T3	0.073 (0.107 to 0.252)	0.075		0.988
		T2 to T3	0,225* (0.080 to 0.370)	0.060	**	0.001
	Time*Sex (Girls)	T1 to T2	-0.101 (-0.388 to 0.185)	0.119		>.99
		T1 to T3	0.201 (-0.069 to 0.471)	0.112		0.221

		T2 to T3	0,303* (0.085 to 0.521)	0.091	**	0.003
	Time*Sex (Boys)	T1 to T2	-0.202 (-0.453 to 0.049)	0.104		0.160
		T1 to T3	-0.055 (-0.292 to 0.181)	0.098		>.99
		T2 to T3	0.147 (-0.044 to 0.338)	0.079		0.196
	Time*Sports Club (Yes)	T1 to T2	-0.267 (-0.559 to 0.026)	0.121		0.087
		T1 to T3	0.109 (0.166 to 0.384)	0.114		>.99
		T2 to T3	0,376* (0.153 to 0.598)	0.092	***	<.001
	Time*Sports Club (No)	T1 to T2	-0.037 (-0.282 to 0.207)	0.102		>.99
		T1 to T3	0.037 (-0.194 to 0.267)	0.096		>.99
		T2 to T3	0.074 (-0.112 to 0.260)	0.077		>.99
VSR SDS (MAKFIT)	Time	T1 to T2	-0.009 (-0.136 to 0.119)	0.053		>.99
		T1 to T3	0,184* (0.055 to 0.314)	0.054	**	0.002
		T2 to T3	0,193* (0.065 to 0.321)	0.053	**	0.001
	Time*Sex (Girls)	T1 to T2	0.004 (-0.188 to 0.196)	0.080		>.99
		T1 to T3	0,304* (0.109 to 0.499)	0.081	**	0.001
		T2 to T3	0,300* (0.107 to 0.493)	0.080	**	0.001
	Time*Sex (Boys)	T1 to T2	-0.021 (-0.189 to 0.146)	0.070		>.99
		T1 to T3	0.064 (-0.107 to 0.235)	0.071		>.99
		T2 to T3	0.085 (-0.083 to 0.254)	0.070		0.671
	Time*Sports Club (Yes)	T1 to T2	-0.005 (-0.200 to 0.191)	0.081		>.99
		T1 to T3	0,236* (0.037 to 0.435)	0.083	*	0.014
		T2 to T3	0,241* (0.044 to 0.437)	0.082	*	0.010
	Time*Sports Club (No)	T1 to T2	-0.013 (-0.176 to 0.151)	0.068		>.99
		T1 to T3	0.132 (-0.034 to 0.298)	0.069		0.169
		T2 to T3	0.145 (-0.020 to 0.309)	0.068		0.104
RD z-value (Fetz)	Time	T1 to T2	0,275* (0.053 to 0.497)	0.092	**	0.009
		T1 to T3	0.040 (-0.186 to 0.266)	0.094		>.99
		T2 to T3	-0,235* (-0.431 to -0.039)	0.081	*	0.012
	Time*Sex (Girls)	T1 to T2	0.222 (-0.112 to 0.557)	0.139		0.332
		T1 to T3	0.055 (-0.286 to 0.395)	0.141		>.99
		T2 to T3	-0.168 (-0.463 to 0.127)	0.122		0.513
	Time*Sex (Boys)	T1 to T2	0,328* (0.034 to 0.621)	0.122	*	0.023
		T1 to T3	0.025 (-0.273 to 0.323)	0.124		>.99
		T2 to T3	-0,302* (-0.560 to -0.044)	0.107	*	0.015
	Time*Sports Club (Yes)	T1 to T2	0.124 (-0.218 to 0.465)	0.142		>.99
		T1 to T3	-0.081 (-0.428 to 0.266)	0.144		>.99
		T2 to T3	-0.205 (-0.505 to 0.096)	0.125		0.306
	Time*Sports Club (No)	T1 to T2	0,426* (0.141 to 0.712)	0.119	**	0.001
		T1 to T3	0.161 (-0.130 to 0.451)	0.121		0.550
		T2 to T3	-0,266* (-0.517 to -0.014)	0.104	*	0.034

a = adjusted for multiple comparisons using Bonferroni correction. p-lvl (*p*-value level) * = $p < 0.05$, ** = $p < 0.01$, *** = $p < 0.001$, BMI = body mass index, CI = confidence interval, IOTF = BMI SDS based on International Obesity Taskforce reference centile curves [7], SD = standard deviation; SDS = standard deviation score, z-value = traditional z-score standardization; 6MR = 6 minute run, SLJ = standing long jump, MB1kg = medicine ball throw (1 kilogram), 4 x 10 SHR

= 4-m x 10-m shuttle run, JS = jumping sideways, VSR = V sit-and-reach Test, RD = ruler drop test, m = meter, SDS = standard deviation score, z-value = traditional z-score standardization; DüMo = Düsseldorfer Modell [1], KATS-K = Karlsruher test system [2], MCA = Motor Competence Assessment [3], MoMo = Motor Module [4], MAKFIT = Macedonian fitness meter [5], F&K = Sport motor tests by Fetz and Kornexl [6], T1 = baseline measurements in September 2019, T2 = follow-up measurements in September 2020, T3 = follow-up measurements in September 2021.

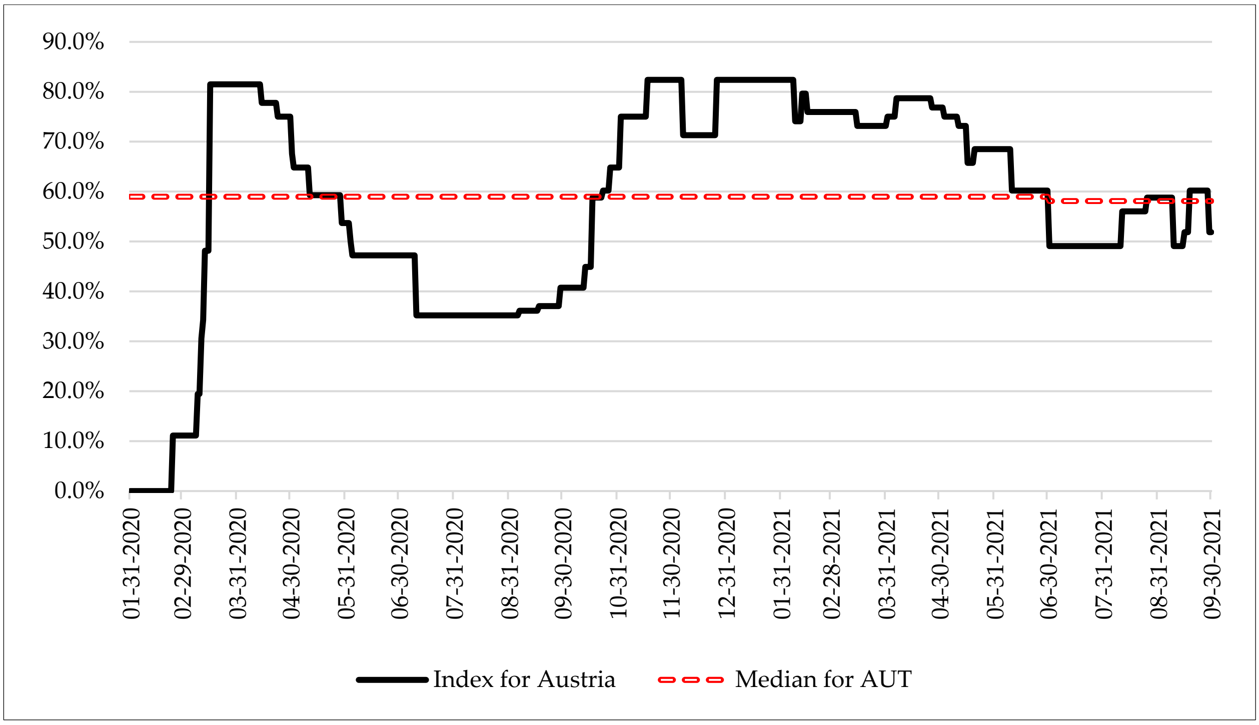

Figure S1. OxCGRT Stringency Index; Figure S1 shows the Oxford COVID-19 Government Response Tracker (OxCGRT) Stringency Index for Austria between January 31, 2020 and September 30, 2021.

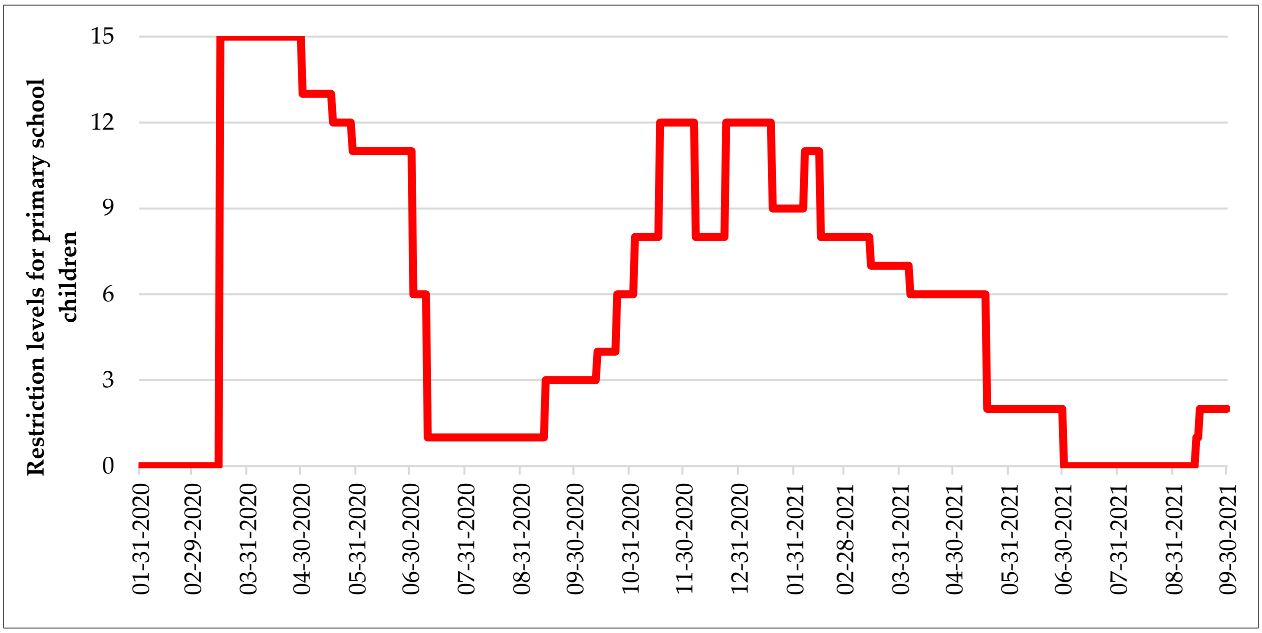

Figure S2. Restriction levels for primary school children; The detailed description of this classification method used in Figure S2 is available in Table S1.

References

1. Stemper, T.; Bachmann, C.; Diehlmann, K.; Kemper, B. *DüMo Düsseldorfer Modell der Bewegungs-, Sport- und Talentförderung*: *2003 - 2018: Konzept, Normwerte, Untersuchungsergebnisse;* LIT: Berlin, 2020, ISBN 9783643147783.
2. Bös, K.; Opper, E.; Woll, A.; Liebisch, R.; Breithecker, D.; Kremer, B. Das Karlsruher Testsystem für Kinder (KATS-K) - Testmanual. *Haltung und Bewegung* **2001**, *21*, 4.
3. Rodrigues, L.P.; Luz, C.; Cordovil, R.; Bezerra, P.; Silva, B.; Camões, M.; Lima, R. Normative values of the motor competence assessment (MCA) from 3 to 23 years of age. *J. Sci. Med. Sport* **2019**, *22*, 1038–1043, doi:10.1016/j.jsams.2019.05.009.
4. Bös, K.; Worth, A.; Heel, J.; Opper, E.; Romahn, N.; Tittlbach, S.; Wank, V.; Woll, A. Testmanual des Motorik-Moduls im Rahmen des Kinder- und Jugendgesundheitssurveys des Robert Koch Instituts. *Haltung und Bewegung (ISSN 0178-3033)* **2004**, *24*.
5. Gontarev, S.; Kalac, R.; Velickovska, L.A.; Zivkovic, V. Estándares de referencia de aptitud física en niños y adolescentes de Macedonia: el estudio MAKFIT. *NUTRICION HOSPITALARIA* **2018**, *35*, 1275–1286, doi:10.20960/nh.1881.
6. Fetz, F.; Kornexl, E. *Sportmotorische Tests*: *Praktische Anleitung zu sportmotorischen Tests in Schule und Verein*, 2., überarb. u. erw. Aufl.; Österr. Bundesverl.: Wien, 1978, ISBN 387039028X.
7. Cole, T.J.; Lobstein, T. Extended international (IOTF) body mass index cut-offs for thinness, overweight and obesity. *Pediatr. Obes.* **2012**, *7*, 284–294, doi:10.1111/j.2047-6310.2012.00064.x.
